# Reduced immunogenicity of MYC amplified, metastatic prostate cancer

**DOI:** 10.18632/oncoscience.644

**Published:** 2026-02-07

**Authors:** Sunny Kahlon, Vayda R. Barker, Mallika Varkhedi, Alex Y. Wang, Taha I. Huda, George Blanck

**Affiliations:** ^1^Department of Molecular Medicine, Morsani College of Medicine, University of South Florida, Tampa, FL 33612, USA; ^2^Department of Immunology, H. Lee Moffitt Cancer Center and Research Institute, Tampa, FL 33612, USA

**Keywords:** prostate cancer, MYC amplification, adaptive immune receptor recombinations, reduced immunogenicity, RNAseq files

## Abstract

Objectives: Through a genomics-based approach analyzing gene expression levels and adaptive immune receptor recombinations, we sought to determine whether MYC amplification was associated with a worse outcome and reduced immunogenicity.

Methods: MYC copy numbers and the presence of adaptive immune receptor (IR) recombination sequencing reads were quantified in genomics files representing prostate cancer samples.

Results: Our results showed that increased MYC amplification was found in metastatic stages of prostate cancer. Furthermore, increased MYC amplification was not only associated with worse progression-free survival but also with reduced immunogenicity in metastatic tumors, as determined by the recovery of a reduced numbers of adaptive IR recombination sequencing reads from tumor RNAseq and tumor whole genome sequence files.

Conclusions: MYC amplification is associated with reduced tumor immunogenicity as assessed by the recovery of IR recombination reads from prostate cancer genomics files.

## INTRODUCTION

Prostate cancer remains a predominant health challenge globally, marked by its status as one of the most common types of cancer among men. The incidence of prostate cancer varies significantly across different regions, reflecting a complex interplay of genetic, environmental, and lifestyle factors [1]. Despite advancements in screening and treatment strategies, prostate cancer continues to impose a significant burden, with varying prognoses depending on the stage at diagnosis and the molecular features of the tumor [2]. The spectrum of prostate cancer disease extends from localized primary tumors, which often have a high survival rate, to advanced metastatic disease with worse survival outcomes, necessitating more comprehensive therapeutic approaches [3].

Oncogenes such as MYC, ERG, and AKT1 impact apoptosis and a number of cell cycle regulatory pathways [4–6]. Furthermore, the interaction between tumor cells and the immune system, especially how tumors evade immune surveillance via immune checkpoints, has become an important issue for therapy [7]. These two factors, oncogenes and prostate cancer immunology, in particular, reflect the disease’s heterogeneity and the prospects for personalized therapy.

More specifically, it has been known for some time that neuroblastoma tumors with amplification of MYCN are immunologically cold [8, 9]. Recently, this conclusion has been validated and extended with the assessment of adaptive immune receptor (IR) recombination reads in MYCN amplified versus non-amplified neuroblastoma tumors, from two independent neuroblastoma datasets [10]. Also, the approach of assessing the IR recombination reads led to a greater specificity with regard to which arm of the adaptive immune system appeared to be in deficit, with results indicating that MYCN amplified neuroblastomas evidence only reduced numbers of T-cells whereas EGFR amplified glioblastoma samples have reduced numbers of both T- and B-cells but not reduced numbers of gamma-delta T-cells [10]. Thus, we evaluated the relationship between MYC amplification and detection of adaptive IR recombinations for prostate cancer.

## RESULTS

### MYC CNV in primary and metastatic datasets

**Figure 1:**
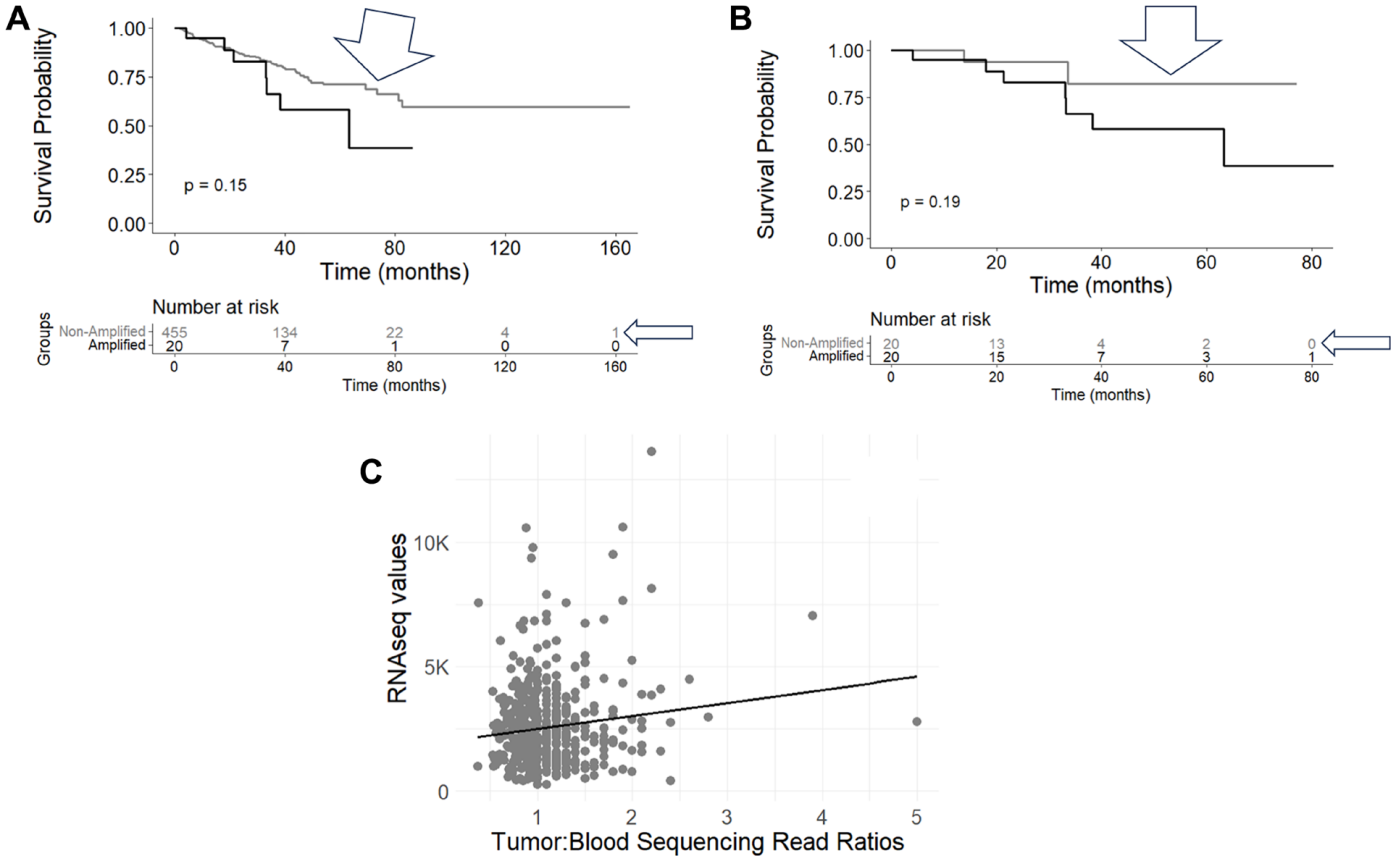
Kaplan-Meier (KM) survival and gene expression analyses of TCGA case IDs representing MYC amplification. (**A**) Progression-Free Survival (PFS) of case IDs representing WXS-based CNs of MYC, comparing top 20 CNV ratios (black line, *n* = 20) and all other CNV ratios (grey line, arrowheads, *n* = 455), with statistical analysis for the KM plot and the 70-month survival timepoint (log-rank *p*-value = 0.15; two-proportion time, single time point test *p*-value = 0.0113) (Supplementary Table 4). (**B**) PFS of case IDs representing WXS-based CNs of MYC, comparing top 20 CNV ratios (blue line, *n* = 20) and bottom 20 CNV ratios (gray line, *n* = 20), with statistical analysis for the KM plot and the 70-month survival timepoint (log-rank *p*-value = 0.19; two-proportion time, single time point test *p*-values = 0.0057) (Supplementary Table 5), (**C**) Correlation of MYC tumor:blood sequencing read ratios (CNV) with the RNAseq values for MYC (r = 0.128, *p* = 0.005) (Supplementary Table 6).

**Figure 2:**
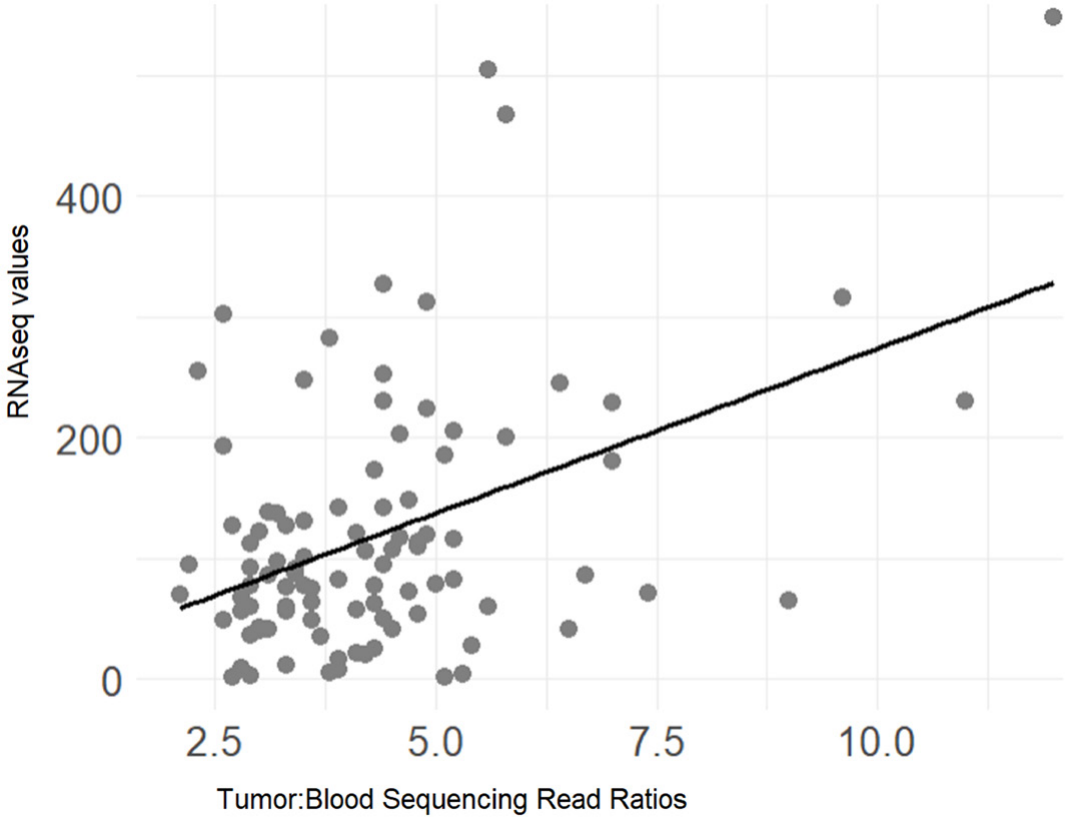
Correlation of RNAseq values with tumor: blood sequencing read ratios for MYC, representing case IDs from the WCDT-MCRPC dataset. (r = 0.442, *p* < 0.001) (Supplementary Table 6).

To assess potential survival probability distinctions based on the amplification of oncogenes, we first assessed the CNV for several oncogenes using the precision-guided method of Mauro, Varkhedi and colleagues [11, 12], whereby we established ratios of tumor and blood read counts for each case ID in the TCGA-PRAD and WCDT-MCRPC datasets (Methods). For the TCGA-PRAD dataset, the case IDs representing the top 20 read count ratios versus all remaining cases (representing all of the lower read count ratios) were assessed with a KM analysis for progression-free survival (PFS). In the case of MYC CNV, a survival difference at the 70-month timepoint was observed, where the cases with a greater number of MYC copies represented a lower PFS probability (Figure 1A, two-proportion test *p*-value = 0.0113). When comparing the cases representing the top 20 and bottom 20 read count ratios with a KM analysis, a similar result was seen at the 70-month timepoint, where the cases with a greater number of MYC copies represented a worse PFS (Figure 1B, two-proportion test *p*-value = 0.0057). We next assessed whether the apparent MYC copy numbers (CNs) correlated with MYC gene expression levels, for the TCGA-PRAD dataset, which revealed a statistically significant positive correlation for the MYC CNs and the MYC RNAseq values (Figure 1C, Pearson’s correlation coefficient, R = 0.128, Pearson’s correlation *p*-value = 0.005). The assessment of a correlation of MYC copies and MYC RNAseq values was also performed for the WCDT-MCRPC dataset (Methods), which revealed a significant positive correlation for the MYC CNs and the MYC RNAseq values (Figure 2, Pearson’s correlation coefficient, R = 0.442, Pearson’s correlation *p*-value < 0.001).

### MYC CN ranges compared across different datasets, to assess potential differences in amplification levels across different stages of prostate cancer

The WCDT-MCRPC dataset, representing metastatic prostate cancer, exhibited a significantly higher average MYC copy number compared to the TCGA-PRAD dataset, which represents primary prostate cancer (Table 1, *p* < 0.001). Similarly, the CMI-MPC dataset (Methods), also representing metastatic prostate cancer, demonstrated a notably elevated average MYC copy number when contrasted with the TCGA-PRAD dataset (Table 1, *p* < 0.001).

**Table 1:** Average MYC copy numbers for three prostate cancer datasets as determined by the approach of Varkhedi et al. [12]

Dataset	Mean tumor: blood sequencing read ratio	STDev tumor: blood sequencing read ratio	*p*-value: comparison with TCGA-PRAD
TCGA-PRAD	1.14	0.42	−
WCDT-MCRPC	4.32	1.71	<0.0001
CMI-MPC	3.31	2.35	<0.0001

See Methods and SOM Supplementary Tables 1–3.

### Recovery of adaptive IR recombination reads from MYC amplified and non-amplified cases

To determine whether there was a difference in the recovery of adaptive IR recombination reads for MYC-amplified versus nonamplified prostate cancer, the immunoglobulin heavy (IGH) and light (IGK, IGL) chain recombination reads from exome and RNAseq files were extracted from the TCGA-PRAD dataset (Methods) [13–15]. For these preliminary considerations, average recombination read counts for the MYC-amplified cases were not significantly different than for MYC-nonamplified cases (data not shown). This process was repeated for the WCDT-MCRPC dataset, for both WGS and RNAseq files. A cutoff tumor:blood sequencing read ratio of ≥5.0 was used to distinguish MYC amplified from non-amplified cases. For the RNAseq file-derived recombination reads, MYC amplified cases represented significantly reduced number of IGH recombination reads (*p* = 0.0056), IGK recombination reads (*p* = 0.0149), IGL recombination reads (*p* = 0.0039), and IGH+IGK+IGL recombination reads combined (*p* = 0.0021) (Table 2). For WGS file-derived recombination reads, cases with MYC amplification represented significantly reduced numbers of IGK recombination reads (*p* = 0.0238), IGL recombination reads (*p* = 0.0011), and IGH + IGK + IGL recombination reads combined (*p* = 0.0024) (Table 2).

**Table 2:** Summary of adaptive IR recombination read counts and p-values for case IDs of WCDT- MCRPC dataset representing either amplified or non-amplified MYC genes, respectively

Dataset	WCDT-MCRPC RNA_Seq Files (a, b)	WCDT-MCRPC WGS Files (a, b)
**Mean IGH count, MYC non-amplified cases**	1122.80 (61 cases)	1.88 (9 cases)
**Mean IGH count, MYC amplified cases**	33.27 (15)	0
***t*-test p-value**	0.0056	0.0122
**Mean IGK count, MYC non-amplified cases**	884.25 (67)	1.33 (12)
**Mean IGK count, MYC amplified cases**	40.93 (15)	0
***t*-test p-value**	0.0149	0.0013
**Mean IGL count, MYC non-amplified cases**	2082.44 (63)	1.62 (21)
**Mean IGL count, MYC amplified cases**	88.27 (15)	1.00 (2)
***t*-test p-value**	0.0039	0.0040
**Mean combined IG count, MYC non-amplified cases**	3752.61 (69)	2.31 (29)
**Mean combined IG count, MYC amplified cases**	128.26 (19)	1.00 (2)
***t*-test p-value**	0.0021	0.0001

(a) Case numbers, in parentheses, do not include any cases that had zero adaptive IR recombination read recoveries. (b) A read count ratio of tumor: blood ≥5.0 was classified as gene amplification.

Next, the T-cell receptor alpha (TRA), beta (TRB), gamma (TRG), and delta (TRD) recombination read counts from WGS and RNAseq files were extracted from the TCGA-PRAD dataset. For all these comparisons, average recombination read counts for the MYC-amplified cases were not significantly different than for MYC-nonamplified cases (data not shown). This process was repeated for the WCDT-MCRPC dataset (Methods), for both WGS and RNAseq files. A cutoff tumor:blood sequencing read ratio of ≥5.0 was used to distinguish MYC amplified from non-amplified cases, as in Table 2. For WGS file-derived recombination reads, cases with MYC amplification revealed significantly reduced numbers of TRA recombination reads (*p* = 0.0077), TRB recombination reads (*p* = 0.0167), and TRA + TRB recombination reads combined (*p* = 0.0073) (Table 3). No significant difference was detected in recombination read counts for RNAseq files (data not shown). For all comparisons, there was no significant difference detected in TRG or TRD recombination read counts.

**Table 3:** TCR recombination read counts for case IDs of WCDT- MCRPC dataset, representing either amplified or non-amplified MYC genes

Dataset	WCDT-MCRPC WGS Files
**Mean TRA Count, MYC non-amplified cases**	4.98 (41 cases)
**Mean TRA Count, MYC amplified cases**	2.57 (7 cases)
***t*-test *p*-value**	0.0077
**Mean TRB Count, MYC non-amplified cases**	1.61 (40)
**Mean TRB Count, MYC amplified cases**	0.54 (4)
***t*-test *p*-value**	0.0167
**Mean TRA + TRB Combined Count, MYC non-amplified cases**	6.04 (54)
**Mean TRA + TRB Combined Count, MYC amplified cases**	4.29 (7)
***t*-test *p*-value**	0.0073

Case numbers do not include any cases that had zero adaptive IR recombination read recoveries. A read count ratio of tumor: blood ≥5.0 was classified as MYC gene amplification.

### Expression of an immune signature gene set in MYC amplified and non-amplified cases

**Figure 3:**
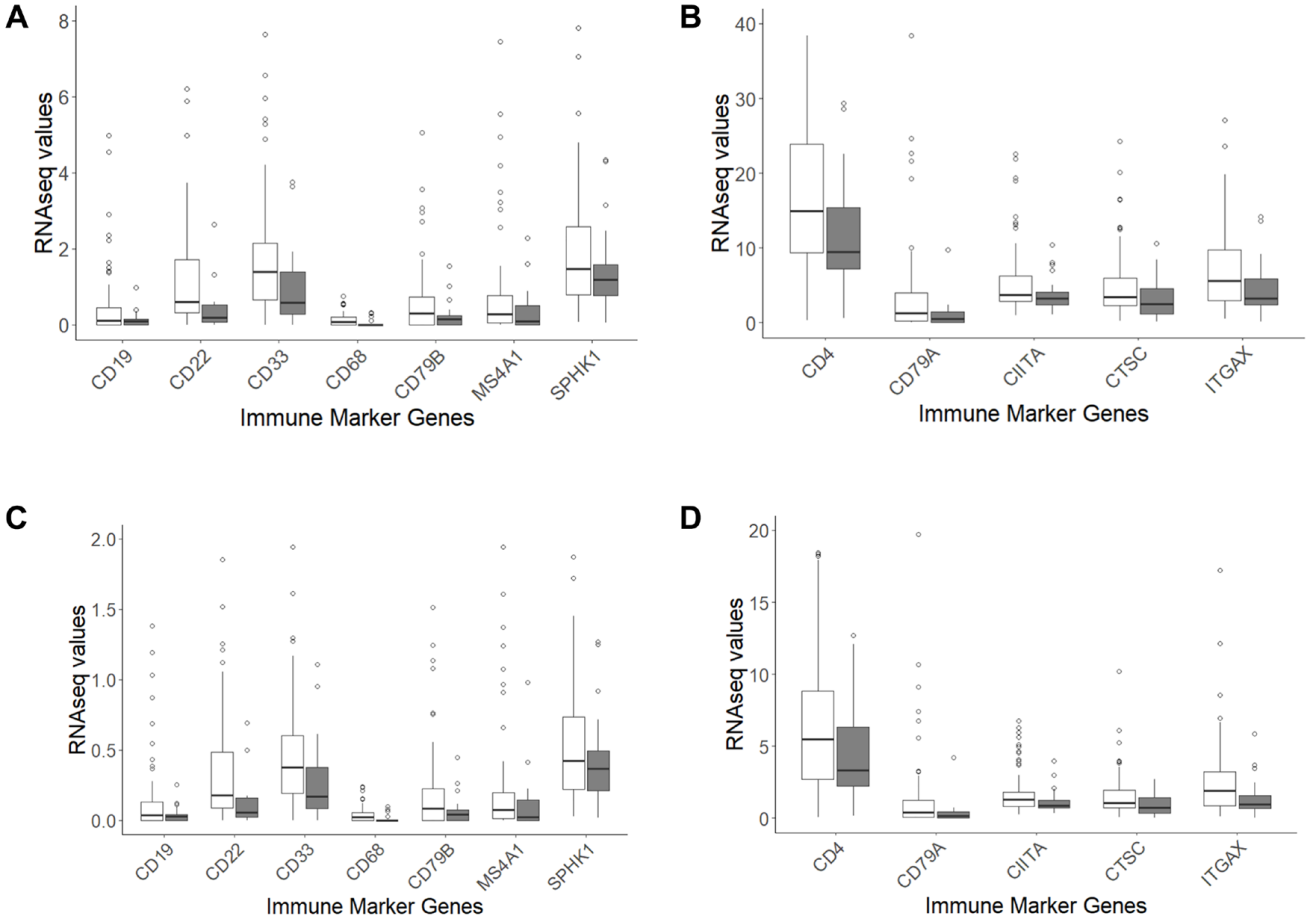
Box and whisker plots of selected immune marker genes for comparison of expression levels (tpm and fpkm) for MYC amplified (shaded) and non-amplified WCDT-MCRPC cases. (**A**) tpm: CD19, *p*-value = 0.004; CD22, *p*-value <0.001; CD33, *p*-value = 0.004; CD68, *p*-value = 0.006; CD79B, *p*-value=0.007; MS4A1, *p*-value = 0.002; SPHK1, *p*-value = 0.037. (**B**) tpm: CD4, *p*-value = 0.038; CD79A, *p*-value = 0.005; CIITA, *p*-value = 0.017; CTSC, *p*-value = 0.018; ITGAX, *p*-value = 0.010. (**C**) fpkm: CD19, *p*-value = 0.002; CD22, *p*-value <0.001; CD33, *p*-value = 0.002; CD68, *p*-value = 0.004; CD79B, *p*-value = 0.004; MS4A1, *p*-value = 0.002; SPHK1, *p*-value = 0.021. (**D**) fpkm: CD4, *p*-value = 0.017; CD79A, *p*-value = 0.007; CIITA, *p*-value = 0.023; CTSC, *p*-value = 0.012; ITGAX, *p*-value = 0.013. For the full range of RNA-seq data corresponding to the specified immune marker genes, along with data for other immune marker genes refer to Supplementary Table 7.

To support the above observations related to the adaptive IR recombinations, the expression of an immune marker gene set [10] was assessed for MYC amplified and non-amplified cases. The RNAseq values for the immune marker gene set for the WCDT-MCRPC MYC amplified and non-amplified cases were extracted (Methods). Both “tpm_unstranded” and “fpkm_unstranded” values were extracted. This approach revealed statistically significantly lower levels of expression of many of the immune marker genes among the MYC amplified cases (Figure 3A–3D and Tables 4, 5).

**Table 4:** Immune Marker Genes with reduced expression in the MYC amplified cases for the WCDT-MCRPC dataset

Immune marker gene	WCDT-MCRPC average RNAseq value for MYC amplified cases	WCDT-MCRPC average RNAseq value for MYC non-amplified cases	Student’s t-test *p*-value
CD33	0.99	2.00	0.004
CD4	15.00	21.77	0.038
CD68	0.04	0.12	0.006
CIITA	3.87	5.74	0.017
ITGAX	4.69	7.86	0.010
CD19	0.14	0.48	0.004
CD22	0.41	1.27	1.76E-04
CD79A	1.13	6.66	0.005
CD79B	0.25	0.62	0.007
MS4A1	0.34	2.56	0.002
CTSC	3.25	5.15	0.018
SPHK1	1.45	2.38	0.037

(All RNAseq values for both MYC amplified and non-amplified cases for both datasets are in Supplementary Table 7. A read count ratio of tumor: blood ≥5.0 represented gene amplification. RNAseq values are tpm_unstranded).

**Table 5:** Immune marker genes with reduced expression in the MYC amplified cases for the WCDT-MCRPC dataset

Immune marker gene	WCDT-MCRPC average RNAseq value for MYC amplified cases	WCDT-MCRPC average RNAseq value for MYC non-amplified cases	Student’s *t*-test *p*-value
CD33	0.28	0.67	0.002
CD4	4.48	6.83	0.017
CD68	0.01	0.04	0.004
CIITA	1.18	1.78	0.023
ITGAX	1.45	2.55	0.013
CD19	0.04	0.16	0.002
CD22	0.12	0.40	1.33E-04
CD79A	0.40	2.16	0.007
CD79B	0.07	0.19	0.004
MS4A1	0.11	0.84	0.002
CTSC	0.97	1.61	0.012
SPHK1	0.43	0.74	0.021

(All RNAseq values for both MYC amplified and non-amplified cases for both datasets are in Supplementary Table 7. A read count ratio of tumor: blood ≥5.0 was classified as gene amplification RNASeq values are fpkm_unstranded).

## DISCUSSION

The above findings reveal a correlation of increased MYC amplification with metastatic stages of prostate cancer. And, the MYC amplification is associated with poorer PFS outcomes and reduced immunogenicity, particularly in the MYC-amplified metastatic tumors. This latter result is demonstrated by the reduced recovery of adaptive IR recombination reads and the decreased expression of immune marker genes for the MYC-amplified cases, supporting the indication of a reduced immune response to the cancer, with evidence that the reduction is most pronounced for B-cells. As best authors are aware, this is the first report of reduced immunogenicity of MYC amplified prostate cancer and the related data are consistent with findings for oncogene amplification in the cases of neuroblastoma and glioblastoma [8–10]. In particular, this report of reduced immunogenicity of MYC-amplified, metastatic prostate cancer was based on a relatively efficient and comprehensive computational genomics approach.

The association of MYC amplification and decreased immunogenicity supports existing studies that have identified MYC as a critical oncogene across various cancers, including its role in immune evasion [16, 17]. Our study extends this knowledge by linking MYC amplification in prostate cancer specifically to both an aggressive disease course and a reduced immune response, suggesting that MYC amplification could serve as an important biomarker for prognosis and immune escape. This possibility aligns with the shift towards personalized medicine in oncology, focusing on treatments that target gene alterations and immune status [18].

In previous literature, increased expression of certain oncogenes, specifically EGFR and MET, has been associated with reduced response to PD-L1 immune checkpoint blockade [19]. Furthermore, alterations to tumor immunity in advanced stages of prostate cancer due to oncogenic signaling pathways can lead to reduced effectivity of these therapies [20, 21]. Building on these ideas, it is possible that MYC overexpression may similarly affect the tumor microenvironment, potentially diminishing the effects of immune checkpoint therapies.

The reduction in adaptive immune receptor recombination reads in MYC-amplified tumors suggests a quantitative loss of tumor-infiltrating T cells. From a therapeutic perspective, such tumors may derive limited benefit from immune checkpoint blockade alone, as releasing inhibitory signals would have little effect in the absence of substantial T-cell infiltration. However, it is also conceivable that the reduction in T-cell numbers arises from immune checkpoint activation early in tumor evolution, leading to T-cell exhaustion or exclusion from the tumor microenvironment. If this is the case, strategies that restore T-cell infiltration or function may help re-sensitize these tumors to immunotherapy. Also, it is worth noting that there was no detectable decrease in TRG or TRD recombination reads with MYC amplification. This was also the case for EGFR amplification in GBM [10], raising the question of the potential value of gamma-delta T-cells in immunotherapy approaches.

The above study has several limitations. First, the work is based on mining of adaptive IR recombination reads from RNAseq files, whereas PCR-based, immune repertoire data may provide for a more comprehensive and more precisely quantitative assessment of the reduced immunogenicity, insofar as the adaptive IR reductions are concerned. Second, it is important to repeat the above assessments with a prospective clinical trial to reduce concerns regarding confounding variables.

## MATERIALS AND METHODS

### Access to genomics files and CNV analyses

Precision-guided copy number variation (CNV) assessments were conducted using the approach outlined by Mauro et al. [22] and further developed by Varkhedi et al. [12]. The cancer genome atlas, prostate adenocarcinoma (TCGA-PRAD, phs000178) dataset, accessed via National Institutes of Health database of genotypes and phenotypes (dbGaP) project approval number 6300, was downloaded to USF research computing. The genomic data commons (GDC, https://gdc.cancer.gov/) download tool was used to facilitate the acquisition of whole exome sequence (WXS), binary alignment map (BAM) format file slices, for both blood and tumor samples, with the file slices representing specific genes. The genes and BAM file slices were delineated by their start and end nucleotide numbers, according to the hg38 version of the human reference genome sequence available at genome.ucsc.edu. A GDC-provided reference file (sample sheet) was used to identify the case ID associated with each WXS BAM file slice, as well as to determine whether the file represented a blood-derived normal or a tumor sample. Utilizing this sample sheet, the files were programmatically renamed to reflect their corresponding case IDs and to indicate whether the files were derived from blood or tumor samples. The number of mapped reads in each file was then extracted using a function from the SAMtools package [23, 24]. Following this, a ratio of tumor to blood read counts was computed from the respective files. These ratios were subsequently compiled and outputted to a csv file for further analysis (Supplementary Tables 1–3). This approach was also applied to the count me in-metastatic prostate cancer (CMI-MPC, phs001939) and West Coast dream team-metastatic castration resistant prostate cancer (WCDT-MCRPC, phs001648) datasets, via dbGaP project approval numbers 25670 and 31203, respectively.

### Sample numbers

TCGA-PRAD dataset, comprising 499 primary prostate tumor samples, was analyzed as described above. The approaches above were also applied to the CMI-MPC dataset, consisting of 63 metastatic prostate cancer samples, and the WCDT-MCRPC dataset, which included 101 metastatic castration-resistant prostate cancer samples.

### Extraction of the adaptive immune receptor (IR) recombination reads from genomics files

The procedure for extracting the IR recombination reads is described in ref. [15] and the latest iteration of the software is freely available at https://github.com/kcios/2021. The entire set of data representing the WCDT-MCRPC dataset, except for the recombination sequencing reads themselves, can be obtained at the following link: https://usf.box.com/s/waqy80pz663wd5lo16midh3qdf4gv11n. (The sequencing reads are controlled access data and can only be accessed by dbGaP approved users.) For the RNAseq-based adaptive IR recombination reads, only the files with the “genomic” suffix were sourced. For the WGS-based adaptive IR recombination reads, only the metastatic tumor files were sourced.

### KM analyses

The data for the survival analyses were obtained from cbioportal.org [25, 26] for TCGA-PRAD dataset (Pancancer). The survival distinctions were assessed using a web tool at cbioportal.org and then verified using R software (version 4.3.2) and the survminer package (Supplementary Tables 4 and 5).

### RNAseq values

RNAseq values for TCGA-PRAD dataset (Pancancer) were obtained from cbioportal.org [16, 17]. RNAseq files for the WCDT-MCRPC dataset were obtained from the GDC. Specific data (tpm_unstranded and fpkm_unstranded values) were extracted using R software (version 4.3.2) (Supplementary Tables 6 and 7).

## SUPPLEMENTARY MATERIALS





## Abbreviations

BAM: binary alignment map
CNV: copy number variation
dbGaP: database of genotypes and phenotypes
GDC: genomic data commons
CMI-MPC: count me in, metastatic prostate cancer
IR: (adaptive) immune receptor
PFS: progression-free survival
TCGA-PRAD: the cancer genome atlas, prostate adenocarcinoma
WCDT_MCRP: West Coast dream team, metastatic castration resistant prostate cancer
WGS: whole genome sequence
WXS: whole exome sequence
