# Supplementary material for: Reduced immunogenicity of MYC amplified, metastatic prostate cancer
Source: Oncoscience. 2026 Feb 7;13:35–43. doi: 10.18632/oncoscience.644 (PMC12931888; doi:10.18632/oncoscience.644)
Supplement: Supplementary file 1 [file oncoscience-13-644-s001.pdf]

Reduced immunogenicity of MYC amplified, metastatic prostate cancer

SUPPLEMENTARY MATERIALS

Supplementary Table 1: TCGA-PRAD MYC ratios. See Supplementary Table 1

Supplementary Table 2: CMI-MPC MYC ratios. See Supplementary Table 2

Supplementary Table 3: WCDT-MCRPC MYC ratios. See Supplementary Table 3

Supplementary Table 4: Figure 1A complete censoring data. See Supplementary Table 4

Supplementary Table 5: Figure 1B complete censoring data. See Supplementary Table 4

Supplementary Table 6: RNaseq values for scatter plots (Figure 1C, Figure 2). See Supplementary Table 6

Supplementary Table 7: RNAseq values for box and whisker plots. See Supplementary Table 7
